# Resistance to Blockchain Adoption in Health Care Organizations: Evidence From a Cross-Sectional Study

**DOI:** 10.2196/77933

**Published:** 2026-04-08

**Authors:** Wilfried Bazomanza Nzabandora, Sehl Mellouli, Rohit Nishant

**Affiliations:** 1 Department of Organizational Information Systems, Faculty of Business Administration, Université Laval, 2325 Rue de la Terrasse, Québec, QC, G1V 0A6, Canada, 1 4184557044; 2 Queen’s Business School, Queen's University Belfast, Belfast, United Kingdom

**Keywords:** blockchain-based health care apps, perceived risk theory, blockchain adoption, blockchain, risks, health care

## Abstract

**Background:**

Traditional health care systems struggle to ensure the security of medical data. To address these issues, organizations are exploring blockchain-based solutions, which offer strong security for managing medical data and transactions. Despite these benefits, adoption remains limited because many health care organizations are hesitant to implement blockchain apps due to perceived risks associated with these apps.

**Objective:**

Our research aims to study the adoption of blockchain-based health care apps in health care organizations by adopting a risk-based approach. Using perceived risk theory (PRT), we developed a research model that links specific perceived risks of blockchain-based health care apps to the intention to adopt them.

**Methods:**

This study used a cross-sectional design to test the research model through an online questionnaire administered to IT professionals from health care organizations in Canada and Africa. IT professionals were selected because they influence technology adoption and possess greater blockchain knowledge than other health care staff. A total of 217 responses were collected; after removing incomplete entries, 194 valid responses remained for analysis. This exceeded the minimum recommended sample size of 100 for structural equation modeling with 8 latent variables, 40 observed variables, a *P* value of .05, and an anticipated effect size of 0.3. Partial least squares structural equation modeling was used to test the hypothesized relationships in the research model.

**Results:**

Harman single-factor test showed that the first factor accounted for 25.376% of the total variance (below 50%), indicating that common method bias was not a concern. Partial least squares structural equation modeling identified key risks influencing the overall perceived risk of blockchain-based health care apps and their adoption in health care organizations. The findings revealed that the relationship between perceived risk and intention to adopt blockchain-based health care apps is significant in Canadian health care organizations but not in African ones. Additionally, the availability of cloud-based blockchain solutions was found to reduce organizations’ perceived risk related to insufficient resources for adopting and implementing blockchain-based health care apps.

**Conclusions:**

This study used PRT to identify risks that may hinder the adoption of blockchain-based health care apps. Two risks—medical data disclosure and loss of control over medical data—were identified as blockchain-specific and incorporated as new PRT dimensions, extending the theory for blockchain contexts. The extended PRT can guide future studies examining reluctance to adopt blockchain apps. Results also show that contextual differences between Canada and Africa influence the relationship between perceived risks and adoption intentions. Our findings are beneficial to governments, health care organizations, and blockchain development agencies, as they will be able to implement strategies to mitigate some of the risks perceived by IT professionals related to blockchain-based health care apps.

## Introduction

### Overview

Over the past decade, different studies have examined the application of blockchain technology in different business domains [1,2]. Some of these studies are conducted in the health care domain [3,4]. The interest in the health care sector is explained by the profound transformations that blockchain can bring to this field concerning the management of medical data [5-7]. The health care sector is a unique context. It is highly regulated, as it includes sensitive data for which confidentiality must be respected [8]. Blockchain-based apps in this domain may provide more protection for health data by ensuring its confidentiality, availability, interoperability, and integrity [9]. Notable examples include (1) Medifakt (AKT Health), an Estonia-based decentralized blockchain platform for health care that integrates internet of things and machine learning technologies to improve patient care, enhance security, increase data transparency, and promote interoperability across health care systems [10]. The platform supports the transfer, storage, interpretation, and artificial intelligence evaluation of medical data and images [11]. (2) Embleema (Robert Chu), a blockchain solution designed to transform clinical trials and regulatory processes by enabling participants to digitally consent to the collection, storage, and analysis of their health care data [12]. This accelerates drug development while ensuring data integrity and transparency throughout the research lifecycle. (3) Blockpharma (Blockpharma), a blockchain-based platform that strengthens transparency, traceability, and security in pharmaceutical supply chain management [13]. Recording each stage of the supply chain enables patients to verify the authenticity of their medication and supports rigorous quality control through real-time monitoring.

Studies on blockchain in the health care sector are mainly focused on identifying its use cases and opportunities [14]. An analysis of these studies shows that blockchain is used in the health care sector for three main purposes: (1) patient sovereignty over their health care data [15], (2) interoperability of electronic health records [16,17], and (3) integrity and audit trail of medical transactions [18]. Besides the studies that identify blockchain’s use cases and opportunities, other studies focus on analyzing the antecedents of the adoption of blockchain-based apps in the health care sector [19]. These studies take a social-cognitive approach, analyzing the factors that would facilitate the adoption of blockchain-based apps at individual or organizational levels [19-24]. Despite these studies, the health care sector is experiencing a low rate of adoption of blockchain-based apps [19]. The low adoption rate of blockchain-based apps in many sectors could be explained by organizations’ reluctance to adopt them due to uncertainty about their risks, financial benefits, and performance [25]. The lack of successful use cases for blockchain-based apps prevents potential adopters from perceiving these risks and benefits [25]. This is the case in the health care sector, where blockchain is still maturing, and its various use cases are only for experimental purposes, with very basic functionalities [26].

Against this background, our study focuses on the low adoption of blockchain-based apps in the health care sector. We adopt a social-cognitive approach, using perceived risk theory as a theoretical lens, to identify how and to what extent certain perceived risks of blockchain-based apps can prevent their adoption in health care organizations. Identifying these risks will help explain why health care organizations’ intentions to adopt blockchain-based apps do not translate into acts of adoption.

### Theoretical Foundation and Conceptual Framework

This study analyzes how health care organizations’ perceptions of the risks associated with blockchain-based apps influence their intention to adopt them. Therefore, we use perceived risk theory as the theoretical lens of our study, as it helps to explain the influence of individuals’ or organizations’ risk perceptions on their intention to adopt an IT.

### Perceived Risk Theory

Developed by Bauer [27], perceived risk theory was initially used in studies interested in analyzing the factors that would explain individuals’ purchasing decisions [28,29]. Unlike other individual behavior analysis theories, such as the technology acceptance model, which focuses on the factors likely to influence behavior [30], perceived risk theory focuses on factors that could explain individuals’ resistance to adopt a particular behavior [31,32]. Thus, this theory establishes a relationship between the perceived risk of a behavior and its adoption by individuals [32].

A perceived risk is defined as a customer’s perception of distrust and potential side effects that could affect the purchase of a product or service [33]. Featherman and Pavlou [34] also define this concept as the feeling of uncertainty regarding possible negative consequences of using a product or service. The literature includes different conceptualizations of perceived risk [35]. In some studies, it is conceptualized as a unidimensional concept [36,37] and in other studies as a multidimensional concept [35,38]. The conceptualization of perceived risk as a multidimensional concept makes it possible to consider it as a construct composed of a certain number of dimensions that represent the types of risks that can influence individuals’ behavior [35,39]. For example, in a research study on online shopping, authors conceptualized perceived risk as an aggregate construct of 6 risk dimensions: financial, performance, social, physical, psychological, and time [40]. In information systems research, authors consider other types of risks, such as privacy and security risks (SRs), which extend the list of perceived risk dimensions [40].

### Conceptual Framework and Research Hypotheses

**Figure 1.**
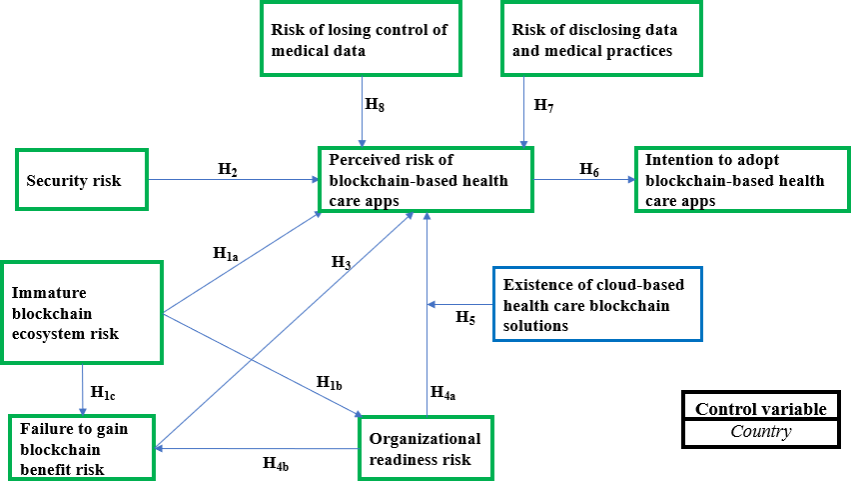
Conceptual framework.

Our conceptual framework includes 6 types of risks that constitute the perceived risk dimensions of blockchain-based health care apps (BBHAs): SR, failure to gain blockchain benefits risk (FGBBR), organizational readiness risk (ORR), risk of losing control of medical data (RLCMD), risk of disclosing data and medical practices (RDDMP), and immature blockchain ecosystem risk (IBER; Figure 1). These risks and the assumptions of our conceptual framework are explained in the following paragraphs.

### IBER in Health Care

The health care blockchain ecosystem is crucial for the adoption of blockchain-based apps and includes adopters, developers, consultants, auditors, regulators, and standardization bodies [41]. Each stakeholder supports adoption by ensuring app availability, guiding implementation decisions, and establishing regulatory frameworks that protect patient data. A mature ecosystem benefits from an increasing number of adopters, enabling partnerships among health care organizations that enhance the quality of health care services [8]. These collaborations encourage knowledge sharing and improve system security through broader participation in network consensus. Consequently, an immature blockchain ecosystem poses a significant risk, increasing health care organizations’ perceived risk when adopting blockchain-based apps. Beyond shaping risk perceptions, the immaturity of the blockchain ecosystem also reduces health care organizations’ readiness to adopt blockchain-based apps [42]. Indeed, the limited availability of blockchain-skilled professionals restricts organizations’ ability to recruit personnel capable of implementing and maintaining blockchain health care systems [43,44]. Second, the small number of adopters limits access to feedback from prior blockchain investment projects [7,45]. Without such feedback, health care organizations lack clear information about resources required for adoption and use. These consequences of blockchain ecosystem immaturity weaken organizational preparedness and help explain the hesitation of many health care organizations to adopt BBHAs. The risk of the blockchain ecosystem’s immaturity can also negatively affect the expected benefits of blockchain-based apps. For example, low blockchain adoption by health care organizations can lead to weak performance of the blockchain network [7]. This weak performance can result from a lack of business partnerships among health care organizations to share medical data through blockchain-based apps [7]. This can negatively impact the expected improvement in health care quality for health organizations.

### SR of Blockchain-Based Apps

The ability of IT to guarantee the security of user data is decisive in the choice of its use at both the individual and organizational levels [40,46]. Health care organizations are driven by the adoption of blockchain-based apps to ensure greater security of patient medical data [47,48]. However, the security flaws identified in current blockchain-based apps indicate that these apps remain vulnerable to security attacks [49]. In BBHAs, several factors can contribute to security flaws, including a lack of patient education [50], poor governance of medical data [48], and noncompliance with regulations [51]. The negative consequences of risks associated with these factors for the security of medical data could influence health care organizations’ perceptions of risk when adopting blockchain-based apps.

### FGBBR

The risk of failure to gain the benefit of a product refers to the possibility that a product does not work as intended and/or does not provide the desired benefits [35]. For BBHAs, the failure to obtain the benefits of their use could be caused by several factors, including the nonintegration between these apps with existing health systems, the resistance of patients to share their medical data, and finally, the lack of alignment between the functionalities of these apps and business needs [7,52]. The risks associated with these factors may prevent health care organizations from fully realizing the benefits of blockchain-based apps, as they could negatively influence health care organizations’ risk perceptions regarding adoption.

### ORR

Organizational readiness is defined as “the availability of specific organizational resources to adopt new IT innovations” [20]. In this study, we examine the influence of ORR on health care organizations’ perceptions of risk related to blockchain-based apps. This risk concerns an organization’s lack of IT expertise, IT infrastructure, and financial resources needed to adopt blockchain-based apps. Therefore, we argue that, in the context of blockchain-based apps, inadequate resources for adoption would increase health care organizations’ perceived risks. In several studies, the lack of organizational readiness is considered a factor that negatively influences the quality of use of a technology, and subsequently, the expected performance or profitability after its adoption [53,54]. For blockchain-based apps, the lack of competent human resources to use and maintain them, as well as the lack of technical infrastructure to support them, might not enable proper use and continuity of use within organizations. This can negatively influence the quality of services offered by blockchain-based apps and could prevent organizations from gaining the benefits of these apps.

Next, we investigate the moderating effect of cloud-based blockchain solutions on the relationship between ORR and the perceived risk of BBHAs. In fact, the adoption of blockchain-based apps by organizations requires adequate human, financial, and material resources to better exploit these apps and benefit from them [20]. Thus, the lack of these resources increases the risk perception of health care organizations regarding adopting blockchain-based apps [20]. To overcome this requirement, organizations use cloud computing for its technological, economic, and operational benefits [20,55]. In the blockchain context, cloud-based solutions would allow health care organizations to use apps based on this technology as Software-as-a-Service (SaaS) [56]. This significantly reduces the perceived risk of health care organizations regarding blockchain-based apps.

### Adoption Intention of BBHAs

IT attributes play a major role in organizational IT adoption intention [57]. Before adopting an innovation, any organization evaluates it on 5 attributes: the complexity of the innovation, its compatibility with the organization, the benefit or advantage that it offers compared to other existing technological choices, and its observability and trialability [57]. In addition to these attributes, the intention to adopt an innovation is also influenced by the risks perceived by potential adopters [31,39,58,59]. Therefore, in the context of blockchain-based app adoption, we also argue that risk perception of these apps could negatively influence health care organizations’ adoption intention.

### RDDMP

The transparency of the blockchain’s ledger ensures that the data recorded on it is visible to authorized network members [60]. For health care structures, ledger transparency can increase the risk of data and medical practice disclosure, with negative consequences for structures that disclose their data and medical practices. For example, when a health structure registers its care protocols on the blockchain ledger, other structures authorized to consult it can learn about those protocols. This can cause a loss of competitive advantage for the structure that discloses its protocols. Thus, the negative consequences associated with the disclosure of data and medical practices raise concerns among health care organizations, increasing their perceived risks of adopting blockchain-based apps.

### RLCMD

Integrating blockchain in the health care sector brings a profound transformation in the management of patient medical data [60,61]. This technology puts the ownership of medical data in the hands of patients and places them at the center of its management [62]. Thus, no health structure can consult or use patients’ medical data without consent [62]. The obligation to obtain patients’ consent before consulting their medical data and the control patients can exercise over how their data are used significantly limit the power of health care structures over patients’ medical data. This limitation on health care organizations’ power over medical data increases their perception of risks regarding adopting blockchain in medical data management.

Each risk described above gives rise to a research hypothesis. Therefore, our research is based on the following hypotheses:

- H1: The risk of immaturity of the blockchain ecosystem in health care is positively associated with the perceived risk of blockchain-based apps by health care organizations (H1a), the risk of organizational readiness (H1b), and the risk of failing to benefit from blockchain-based apps (H1c).
- H2: SR is positively associated with the perceived risk of blockchain-based apps by health care organizations.
- H3: The risk of failure to gain benefits from blockchain-based apps is positively associated with health care organizations’ perceived risk regarding these apps.
- H4: ORR is positively associated with the perceived risk of blockchain-based apps by health care organizations (H4a), as well as the risk of failure to gain benefits of blockchain-based apps (H4b).
- H5: The existence of cloud-based blockchain solutions (ECBS) moderates the relationship between ORR and the perceived risk of BBHAs, such that the positive relationship between ORR and the perceived risk of BBHAs is weak when cloud-based blockchain solutions are present.
- H6: Perceived risk of blockchain-based apps is negatively associated with the health care organizations’ intention to adopt these apps.
- H7: RDDMP is positively associated with the perceived risk of blockchain-based apps by health care organizations.
- H8: RLCMD is positively associated with the perceived risk of blockchain-based apps by health care organizations.

## Methods

### Overview

In this section, we present the research design for our study, which includes 3 sections: research strategy and questionnaire design, data collection, and common method bias.

### Research Strategy and Questionnaire Design

Our study adopts a cross-sectional research design, as it analyzes data from a population at a specific point in time [63]. Therefore, it was reported in accordance with the STROBE (Strengthening the Reporting of Observational Studies in Epidemiology) guidelines adapted for cross-sectional studies [64]. The dimensions of the perceived risk and their reflexive measures are treated as latent (constructs) and manifest variables (items), respectively. To ensure construct validity, the items for each construct are taken from the literature, which we have subsequently adapted to our research context. We converted all the items to a semantic differential scale (1-10) to minimize common method bias by drawing inspiration from the measurement model proposed by Mitchell [65]. Table 1 shows the definition of constructs and the sources of their items. Table S1 in the Multimedia Appendix 1 provides the questionnaire.

**Table 1.** Operationalization of variables.

Construct and subconstructs (items)	Construct and subconstruct definitions	Sources of items
Intention to adopt		
None	A measure of the strength of one’s intention to perform a behavior (behavioral intention) [66]	Venkatesh et al [67]; Glover and Benbasat [35]; and Teo et al [68]
Perceived risk: the feeling of uncertainty regarding possible negative consequences of using a product or a service [34]		
Failure to gain blockchain benefits risks	Failure to gain blockchain benefits regarding quality health care services, reduced cost, efficiency and security, and regulatory compliance [69]	Garg et al [69]
Security risk	Concerns that data and information may be open to security concerns, such as hacking, inaccurate information dispersal, and access to sensitive information [70]	Shah et al [71]; Bélanger and Carter [72]; and Hartono et al [73]
Organizational readiness risk	Risk beliefs of the health care organization’s lack of financial and technological resources to adopt blockchain-based apps [20,74]	Kouhizadeh et al [70]
Immaturity blockchain ecosystem risk	Lack of community of players, such as blockchain experts and developers, blockchain adopters, and regulators interacting in the health care industry [75]	Alzahrani et al [76]; Kyriakidou et al [77]
Risk of losing control of medical data	Risk beliefs of health professionals regarding the loss of autonomy in managing patients’ medical data [78]	Nogueira et al [79]; Ligiane Cristina Braga de Oliveira et al [80]
Risk of disclosing data and medical practices	Risk beliefs about submitting data and medical practices on the blockchain ledger shared by multiple health care organizations [81]	Bansal et al [82]; Bansal et al [83]

### Data Collection

We conducted a survey to collect data over a 2-month period. This survey used an online questionnaire. Our study used purposive sampling. This type of sampling allowed us to select a sample from which the most can be learned [84]. Thus, IT professionals from health care facilities were selected as respondents to our study because of their greater knowledge of blockchain, a technology central to our research. The choice of a sample composed of IT professionals from health care facilities in Canada and emerging countries (the Democratic Republic of Congo, Burundi, and Rwanda) enabled us to minimize the risk of a low response rate, which could be caused by the nonparticipation of some respondents in our survey due to their ignorance of blockchain. We contacted 65 health care structures by email to request their participation (55 in Africa and 10 in Canada). At the end of the survey, we received 217 responses. After deleting responses with missing values (23), 194 valid responses were used for data analysis. We excluded responses with missing data for 2 primary reasons. First, these cases contained only demographic information and lacked any data on the study variables, making them unsuitable for analysis. Second, the number of excluded responses was minimal (23/243), representing less than 10% of the sample. Therefore, their removal is unlikely to affect the validity or representativeness of the dataset. The valid responses obtained for the study (n=194) exceeded the minimum recommended sample size (n=100) for structural equation modeling with 8 latent variables, 40 observed variables, *P* value of .05, and anticipated effect size of 0.3 [85]. Thus, the sample size was considered adequate for analyzing the influence of IT professionals’ perception of risk regarding blockchain-based apps on their intention to adopt in health care structures. The respondents’ demographic information is shown in Table 2.

**Table 2.** Demographic information.

Variables and categories	Participants, n (%)
Age (years)	
18‐30	56 (28.9)
31‐45	91 (46.9)
46‐60	42 (21.6)
>60	5 (2.6)
Sex	
Female	67 (34.5)
Male	127 (65.5)
Continent	
Africa	189 (97.4)
America	5 (2.6)
Experience with blockchain-based apps	
Yes	171 (88.1)
No	23 (11.9)
Use of blockchain-based app within health care facility	
Yes	1 (0.5)
No	193 (99.5)

### Control Variable

The model estimation includes a control variable, namely country (Figure 1), which we transformed into a binary variable (0=Africa and 1=Canada) before its use in SmartPLS (SmartPLS GmbH). A country’s economic, legal, and political contexts play an important role in the assimilation of innovations within organizations [86]. Therefore, there is a significant difference in the assimilation of innovations between organizations in developed and developing countries [86,87]. Unlike developing countries, developed countries are characterized by an economic, political, and regulatory environment favorable to the assimilation of innovations [86]. Given that our study is conducted in Canada and Africa, we also want to analyze how the difference in context (economic, legal, and political) between Canada and African countries could affect the associations between the variables in the model, depending on whether the health care structures are in Canada or Africa. This justifies the introduction of the country as a control variable in the estimation of our theoretical model.

### Common Method Bias

Common method bias results “from the fact that the predictor and criterion variables are obtained from the same source or rater, whereas others are produced by the measurement items themselves, the context of the items within the measurement instrument, and/or the context in which the measures are obtained*”* [88]. Since this research involved self-reported responses collected from a single setting, we will follow the 3-step approach proposed by Guan et al [89] to address the common method bias. First, the measurement items were adopted from well-established existing research. Second, in the survey, we made it clear to all the respondents that there was no right or wrong answer, and they should choose the answers that they considered the most appropriate. We also highlighted the fact that respondents’ answers would be kept anonymous. Third, we conducted Harman single-factor test by entering all the measurement variables in an exploratory factor analysis using IBM SPSS 22.0. The sample would have a common method bias problem if a single construct explained more than 50% of the extracted variance. The results of the exploratory factor analysis show that the first factor explained 25.376% of the total variance (less than 50%), indicating that common method bias was not a problem in this dataset.

### Ethical Considerations

This research has received ethical approval from the Research Ethics Committee of Université Laval (approval number: 2022‐427 A-1 R-1/11-12-2023). During the data collection phase, each participant was informed of the research objectives and associated risks. This process enabled participants to provide informed consent by signing the designated consent form. To ensure confidentiality and privacy, data were initially collected using the LimeSurvey platform (LimeSurvey GmbH), which stores survey responses on a server located in Canada. Subsequently, the exported data were securely stored on Université Laval’s licensed Microsoft 365 OneDrive server. Participant privacy was further protected by presenting research findings using anonymized data, which prevents the identification of individual respondents. No identification of individual participants is possible in the images of the manuscript or supplementary material. Participation in the study was entirely voluntary and was not contingent upon any monetary or other form of compensation.

## Results

### Overview

We used the partial least squares structural equation modeling (PLS-SEM) method to assess the hypothesized relationships between our constructs (using SmartPLS 4.1.0.8; SmartPLS GmbH) [90]. We follow a 2-phase approach to analyze the PLS-SEM model proposed by Guan et al [89]: (1) evaluation of the reliability and validity of the measurement model and (2) evaluation of the structural model.

### Measurement Model

Table 3 shows the results of the measurement model. The measurement model was tested by examining both convergent and discriminant validity [89]. For testing convergent validity, we assess both item reliability and construct reliability of the measurement model [29]. Throughout the assessment of item reliability, we removed items associated with constructs with factor loading (λ) values below .7. As a result, we deleted 1 RLCMD item (RLCMD1), 2 SR items (SR1 and SR2), 3 IBER items (IBER1, IBER2, and IBER3), 2 ORR items (ORR2 and ORR4), and 4 FGBBR items (FGBBR1, FGBBR3, FGBBR8, and FGBBR10). Removing these items from their respective constructs enabled us to retain a measurement model composed of constructs with items whose factor loadings are greater than or equal to .7, confirming the item reliability of the measurement model. Construct reliability was verified by analyzing composite reliability (CR) and Cronbach α values. Each construct has CR and Cronbach α values that exceed the recommended threshold of .7, demonstrating the construct reliability of the measurement model. Furthermore, the average variance extracted (AVE) for each construct exceeds the minimum required value of .50, which confirms the convergent validity of the constructs in the measurement model [91]. Once convergent validity was established, we assessed discriminant validity. The discriminant validity was assessed using the Fornell and Larcker [92] method. As shown in Table 4, the square root of the AVE of each construct was greater than the correlations it shared with other constructs. For example, the square root AVE for FGBBR is .811, and this is greater than the correlation it shares with other constructs. As this was the case with all constructs, discriminant validity was obtained. Overall, the evidence indicates the adequacy of the measurement model. This leads to the next stage of testing the structural model.

**Table 3.** Measurement model results.

Constructs and items	λ^a^	VIF^b^	Cronbach α	CR^c^	AVE^d^
FGBBR^e^			0.896	0.920	0.657
FGBBR2	0.810	2.275			
FGBBR4	0.822	2.533			
FGBBR5	0.836	2.831			
FGBBR6	0.822	2.324			
FGBBR7	0.807	2.369			
FGBBR9	0.765	1.883			
IABHA^f^			0.844	0.896	0.685
IABHA1	0.894	3.213			
IABHA2	0.725	1.546			
IABHA3	0.902	3.327			
IABHA4	0.776	1.704			
IBER^g^			0.877	0.923	0.800
IBER1	0.888	2.811			
IBER3	0.894	2.772			
IBER5	0.901	2.022			
ORR^h^			0.867	0.919	0.790
ORR1	0.910	2.653			
ORR3	0.876	2.144			
ORR5	0.881	2.189			
RDDMP^i^			0.748	0.851	0.656
RDDMP1	0.734	1.728			
RDDMP2	0.804	1.611			
RDDMP3	0.887	2.144			
RLCMD^j^			0.854	0.906	0.764
RLCMD2	0.870	2.550			
RLCMD3	0.819	1.952			
RLCMD4	0.928	2.116			
SR^k^			0.784	0.857	0.600
SR3	0.732	1.612			
SR4	0.722	2.252			
SR5	0.727	2.248			
SR6	0.812	1.867			
SR7	0.838	1.907			

a λ: standardized factor loading.

b VIF: variance inflation factor.

c CR: composite reliability.

d AVE: average variance extracted.

e FGBBR: failure to gain blockchain benefits risk.

f IABHA: adoption intention of blockchain-based health care apps.

g IBER: immature blockchain ecosystem risk.

h ORR: organizational readiness risk.

i RDDMP: risk of disclosing data and medical practices.

j RLCMD: risk of losing control of medical data.

k SR: security risk.

**Table 4.** Results for discriminant validity.

Constructs	FGBBR^a^	IABHA^b^	IBER^c^	ORR^d^	PRBHA^e^	RDDMP^f^	RLCMD^g^	SR^h^
FGBBR	0.811	–0.231	0.439	0.429	0.864	0.217	0.289	0.428
IABHA	–0.231	0.828	–0.056	–0.061	–0.409	–0.232	–0.047	–0.151
IBER	0.439	–0.056	0.894	0.824	0.711	–0.237	0.790	–0.066
ORR	0.429	–0.061	0.824	0.889	0.732	–0.062	0.552	0.163
PRBHA	0.864	–0.409	0.711	0.732	0.511	0.183	0.509	0.455
RDDMP	0.217	–0.232	–0.237	–0.062	0.183	0.81	–0.480	0.459
RLCMD	0.289	-0,047	0.790	0.552	0.509	–0.480	0.874	–0.232
SR	0.428	–0.151	–0.066	0.163	0.455	0.459	–0.232	0.774

a FGBBR: failure to gain blockchain benefits risk.

b IABHA: adoption intention of blockchain-based health care apps.

c IBER: immature blockchain ecosystem risk.

d ORR: organizational readiness risk.

e PRBHA: perceived risk of blockchain-based health care apps.

f RDDMP: risk of disclosing data and medical practices.

g RLCMD: risk of losing control of medical data.

h SR: security risk.

### Structural Model

**Figure 2.**
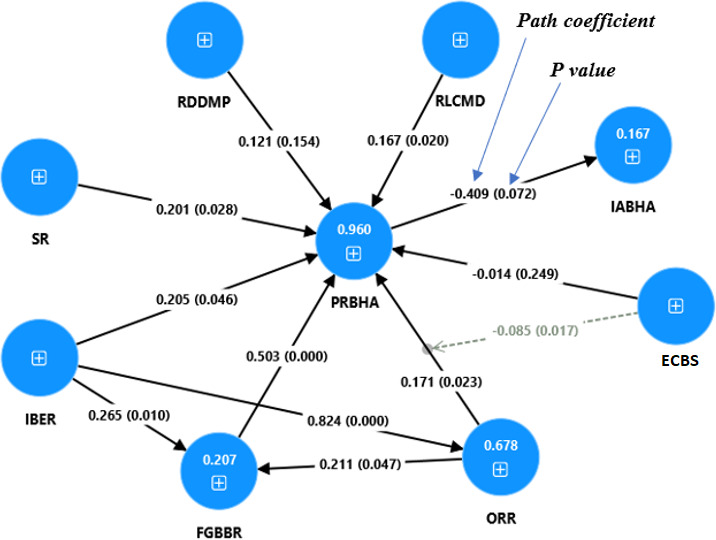
Structural model result. ECBS: existence of cloud-based blockchain solutions; FGBBR: failure to gain blockchain benefits risk; IABHA: adoption intention of blockchain-based health care apps; IBER: immature blockchain ecosystem risk; ORR: organizational readiness risk; PRBHA: perceived risk of blockchain-based health care apps; RDDMP: risk of disclosing data and medical practices; RLCMD: risk of losing control of medical data; SR: security risk.

As the reliability and validity of the variables are established, we examine the structural model to investigate the correlations between the theoretical constructs and test the effect of the moderating variable [93]. The results of the structural model are shown in Table 5 (a summary of the results of hypothesis testing) and Figure 2 (PLS results of the structural model). Before assessing the structural model, we first assess the collinearity issue [94] and the structural model’s predictive accuracy [95]. Table 3 shows that all variance inflation factor values of the items are less than 5, indicating that collinearity is not an issue among the constructs [91]. The measure most often used to assess a structural model’s predictive accuracy in the PLS-SEM method is the *R*^2^ coefficient of determination. Moreover, a model is judged as interesting if it has a sufficiently large *R*^2^ (>0.25) [95]. The results in Table 6 show that 3 constructs (FGBBR: 0.199, ORR: 0.677, and PRBHA [perceived risk of blockchain-based health care apps]: 0.958) are explained at a substantial level and one (IABHA [adoption intention of blockchain-based health care apps]: 0.163) is explained at a weak level [96,97]. Based on the *R*^2^ values, we can state that the model’s predictive accuracy is moderate.

**Table 5.** Structural model results and hypothesis testing.

Hypothesis	Path coefficient	*t* statistics	*P* values	Inference
H_1a_: IBER^a^ → PRBHA^b^	0.205	1.692	.05	Supported
H_1b_: IBER → ORR^c^	0.824	34.743	.00	Supported
H_1c_: IBER → FGBBR^d^	0.265	2.339	.01	Supported
H_2_: SR^e^ → PRBHA	0.201	1.930	.03	Supported
H_3_: FGBBR → PRBHA	0.503	6.129	.00	Supported
H_4a_: ORR → PRBHA	0.171	2.011	.02	Supported
H_4b_: ORR → FGBBR	0.211	1.687	.05	Supported
H_5_: ECBS^f^ x ORR → PRBHA	−0.085	2.133	.02	Supported
H_6_: PRBHA → IABHA^g^	−0.409	1.470	.07	Not supported
H_7_: RDDMP^h^ → PRBHA	0.121	1.021	.15	Not supported
H_8_: RLCMD^i^ → PRBHA	0.167	2.068	.02	Supported

a IBER: immature blockchain ecosystem risk.

b PRBHA: perceived risk of blockchain-based health care apps.

c ORR: organizational readiness risk.

d FGBBR: failure to gain blockchain benefits risk.

e SR: security risk.

f ECBS: existence of cloud-based blockchain solutions.

g IABHA: adoption intention of blockchain-based health care apps.

h RDDMP: risk of disclosing data and medical practices.

i RLCMD: risk of losing control of medical data.

**Table 6.** *R*^2^ values.

Constructs	*R* ^2^	*R*^2^ adjusted	Results
FGBBR^a^	0.207	0.199	Substantial
IABHA^b^	0.167	0.163	Weak
ORR^c^	0.678	0.677	Substantial
PRBHA^d^	0.960	0.958	Substantial

a FGBBR: failure to gain blockchain benefits risk.

b IABHA: adoption intention of blockchain-based health care apps.

c ORR: organizational readiness risk.

d PRBHA: perceived risk of blockchain-based health care apps.

Next, we take a close look at the hypothesized relationships of the structural model. The hypotheses H1a, H2-H4a, and H7-H8 represent the association between PRBHA and its dimensions (IBER, SR, FGBBR, ORR, RDDMP, and RLCMD). Of the 6 dimensions of PRBHA, 5 are positively and significantly associated with PRBHA: RLCMD (*β*=0.167; *P=*.02), SR (*β*=0.201; *P=*.03), IBER (*β*=0.205; *P=*.05), FGBBR (*β*=0.503; *P=.00*), ORR (*β*=0.171; *P=.*02), supporting H1a, H2, H3, H4a, and H8. Contrary to what we assumed, the positive association between RDDMP and PRBHA is not significant (*β*=0.121; *P=*.15). Therefore, hypothesis H7 is not supported. Hypothesis H6 predicted that PRBHA is negatively and significantly associated with IABHA. Contrary to our hypothesis, the negative association between PRBHA and IABHA is not significant (*β*=−0.409; *P=.*07) and does not support H6.

The hypotheses H1b, H1c, and H4b represent the associations between 3 dimensions of perceived risks (IBER, FGBBR, and ORR). As hypothesized, IBER is positively and significantly associated with FGBBR (*β*=0.265; *P=.*01), supporting H1c. Then, IBER is positively and significantly associated with ORR (*β*=0.824; *P=.00*), supporting H1b. Finally, ORR is positively and significantly associated with FGBBR (*β*=0.211; *P=.*05), supporting H4b.

Finally, hypothesis H5 predicts that ECBS moderates the association between ORR and PRBHA. This hypothesis is also supported (*β*=–0.085; *P=.*02), indicating that ECBS dampens the positive association between ORR and PRBHA. In other words, the positive association between ORR and PRBHA is weak when cloud-based blockchain solutions are present.

### Interpretation of the Impact of the Control Variable on the Model Estimation

Tables7  8 present model estimation results without the control variable and with the control variable. Analysis of these results leads to 2 observations. The first observation concerns the association between the control variable (country) and the dependent variable (IABHA). Table 8 shows that this association is not significant (*P*=.34 ). This suggests that whether a health care facility is based in Canada or Africa has no significant effect on that facility’s intention to adopt a BBHA. The second observation concerns the effect of the control variable on the associations between the variables in the model. A comparison of the results in Tables7  8 shows that only one association is affected by the control variable. Unlike the model without the control variable, the association between PRBHA and IABHA becomes significant in the model with the control variable (*P*=.02 ). This means that the perception of the risks of BBHAs does not affect a health care facility’s intention to adopt these apps in the same way, depending on whether the facility is in Canada or Africa.

**Table 7.** Estimation of the model without a control variable.

Associations between variables	Without control variable			
	Original sample (O)	Sample mean (SD)	*t* statistics (O/SD)	*P* values
RDDMP^a^ → PRBHA^b^	0.121	0.125 (0.119)	1.021	.15
ECBS^c^ → PRBHA	−0.014	−0.014 (0.021)	0.679	.25
PRBHA → IABHA^d^	−0.409	−0.339 (0.278)	1.470	.07

a RDDMP: risk of disclosing data and medical practices.

b PRBHA: perceived risk of blockchain-based health care apps.

c ECBS: existence of cloud-based blockchain solutions.

d IABHA: adoption intention of blockchain-based health care apps.

**Table 8.** Estimation of the model with control variable.

Associations between variables	With control variable			
	Original sample (O)	Sample mean (SD)	*t* statistics (O/SD)	*P* values
RDDMP^a^ → PRBHA^b^	0.121	0.144 (0.109)	1.116	.13
ECBS^c^ → PRBHA	−0.014	−0.008 (0.020)	0.703	.24
PRBHA → IABHA^d^	−0.405	−0.436 (0.201)	2.012	.02
Country → IABHA	−0.133	−0.147 (0.319)	0.418	.34

a RDDMP: risk of disclosing data and medical practices.

b PRBHA: perceived risk of blockchain-based health care apps.

c ECBS: existence of cloud-based blockchain solutions.

d IABHA: adoption intention of blockchain-based health care apps.

## Discussion

### Principal Findings

Our study first revealed that the risks of security, failure to obtain blockchain benefits, organizational unpreparedness, disclosure of medical data and practices, and loss of control over medical data increase health care organizations’ risk perceptions regarding BBHAs, reducing their intention to adopt these apps. These findings align with research by Glover and Benbasat [35], which emphasized that the SR of personal data and the failure to gain benefits of a product purchased on the web play an important role in buyers’ overall risk perception regarding electronic transactions. Moreover, our study revealed that the RDDMP does not play a significant role in the overall risk perception of BBHAs. This result aligns with research that views data disclosure via blockchain not as a risk but as an advantage [98]. This is the case with the study by Dubovitskaya et al [98], in which the authors mention that disclosing data via blockchain in biomedical research allows pharmaceutical companies to disclose information about the production process of drugs. This will enable patients and regulatory bodies to know the context in which certain medical products are produced before they are certified or approved.

The second key finding of this study concerns the reciprocal association between 3 perceived risks of BBHAs: IBER, ORR, and FGBBR. The positive association between IBER and ORR indicates that an underdeveloped blockchain ecosystem—characterized by limited expertise in development, governance, and regulatory clarity—heightens the risk of organizational unpreparedness for adopting BBHAs. Additionally, the association between IBER and FGBBR suggests that ecosystem immaturity reduces the likelihood of realizing expected benefits, such as secure medical data sharing. Similarly, ORR is positively associated with FGBBR, as inadequate infrastructure and insufficiently trained personnel compromise the effective use of these technologies. For example, poor cryptographic key management by untrained users can introduce security vulnerabilities in blockchain-based apps [3], thereby preventing organizations from achieving the intended data protection and operational efficiencies of such apps.

The third key finding of our study concerns the association between IT professionals’ perceived risk and their intention to adopt BBHAs. Our results show that organizations perceiving higher risks are less likely to adopt BBHAs, consistent with prior research on IT adoption, including Le et al [99] study on self-service technologies. Their study showed that individual users’ concerns about technological risks reduce their willingness to engage with such systems. However, our study reveals regional differences in the association between perceived risk and intention to adopt BBHAs. In Canada, where health care systems are advanced and heavily funded, organizations are more skeptical due to limited performance feedback on BBHAs, leading to reduced motivation to invest. In contrast, health care organizations in developing African countries, which often have fragmented and underresourced systems, view BBHAs as opportunities to improve service delivery. Thus, perceived risks have a weaker impact on their adoption intentions. This regional variation aligns with the cross-national study by Hoover et al [100] on cultural influences in risk perception and consumer behavior. These authors analyzed the influence of perceived brand risk on purchase intention in Mexico and the United States. They found that the cultural difference between Mexico and the United States makes perceived brand risk a less important factor in purchasing behavior in Mexico than in the United States.

The fourth relevant result of our study comes from the assessment of the moderating effect of the existence of blockchain solutions offered as SaaS on the relationship between organizational unreadiness and overall risk perception regarding BBHAs. The results of our study reveal that the possibility of using BBHAs as SaaS would reduce IT professionals’ perceived risk of the additional human and material resources required for their use and maintenance in health care settings. This result is in line with several studies that have analyzed the factors of cloud computing adoption in organizations [86,101,102]. In their study, Zhu et al [86] mention that the reduction of overall IT costs influences the use of cloud services. These costs include preliminary investment, upgrading, energy saving, and maintenance of cloud services [86,101].

### Theoretical and Practical Contributions

Our study makes a theoretical contribution by adopting and interpreting the perceived risk theory derived from marketing in the context of blockchain adoption in the health care sector. Originally, this theory was used to explain consumers’ purchasing decisions in the context of perceived product or service risks [103-105]. In information systems, perceived risk theory is used to analyze how perceived risks can influence the IT adoption decisions [29,93]. However, this theory addresses risks that apply broadly to IT as a whole, making it unsuitable for analyzing risks within specific IT categories. The results of our study highlight 2 risks specific to blockchain-based apps that are likely to influence the overall perceived risk regarding these apps and, therefore, their adoption in health care structures: RDDMP and RLCMD. Integrating these 2 risks as new constructs into the perceived risk theory contributes to its extension, making it more suitable for risk analysis in blockchain-based apps. Future research analyzing barriers to blockchain adoption in the health care sector can incorporate the 2 new risks into its theoretical model. Moreover, conducting our study in Canada and Africa allowed us to understand how contextual differences between Canada and Africa affect how risks are perceived in BBHAs and the intention to adopt them in Canadian and African health care organizations. This contributed to the generalizability of our results.

As a practical contribution, our study highlights the risks IT professionals in health care organizations perceive regarding BBHAs that may prevent their adoption in health care organizations. Knowledge of these risks would benefit the development agencies of blockchain-based apps and health care organizations [106,107]. For development agencies of blockchain-based apps, our findings will guide their advocacy discourse on the adoption of blockchain-based apps among health care organizations. These agencies can exploit these findings to formulate recommendations to mitigate risks that IT professionals perceive or educate IT professionals of health care organizations on strategies to avoid certain perceived risks [108]. In addition, development agencies of blockchain-based apps can take advantage of our findings to develop BBHAs that minimize some risks that IT professionals perceive regarding these apps. For health care organizations, knowing the risks of BBHAs brings 2 major implications. First, these organizations handle sensitive data that require better protection against security attacks [8]. Through our results, these organizations understand that despite the hype about the security of blockchain-based apps, these apps are exposed to SRs. Therefore, they must implement appropriate security policies to strengthen the security of these apps [107]. Second, our study’s results inform health care organizations’ decisions regarding adopting BBHAs. We believe that a good decision to adopt these apps should consider their benefits and potential risks [109]. Knowing these risks would allow health care organizations to implement strategies to mitigate them and improve the use of BBHAs.

### Limitations

This study has 2 main limitations. The first limitation concerns the small number of IT professionals working in health care structures that have already adopted BBHAs or those in the process of adopting them. In addition, our study had a low participation of IT professionals from health care structures in Canada. The low participation of IT professionals from structures that use BBHAs, structures that are in the process of adopting these apps, and structures in Canada prevents variance in the dependent variable of our study, namely the intention to adopt BBHAs. Second, our research analyzes blockchain adoption in the health care sector, which is experiencing a low adoption rate of BBHAs. All these limitations constitute obstacles to the generalization of the results of this research.

### Directions for Future Research

We propose several avenues for future research. First, the conceptual model was validated using data from IT professionals in health care organizations. This choice was justified by the low knowledge of blockchain among health care professionals and patients [110,111]. It is essential to note that the successful implementation of IT in a health care organization necessitates the involvement of multiple stakeholders, including IT professionals, health care professionals, patients, health care organization managers, and health care regulators [112]. Perceptions of each stakeholder regarding the risks associated with BBHAs may influence health care organizations’ intentions to adopt these apps [113]. Therefore, we recommend that other researchers conduct similar research involving many stakeholders to better assess the factors that influence the intention of health care organizations to adopt BBHAs.

Second, our research focused primarily on the factors that may hinder health care organizations’ intention to adopt blockchain-based apps. Our choice is explained by the low adoption of blockchain-based apps in health care organizations [76]. Therefore, the data for our research were primarily collected from health care organizations that have not yet adopted a blockchain-based app and those that are in the process of doing so. Future research may analyze the adoption challenges of these apps by collecting data from health care organizations that have already adopted these apps. Thus, health care organizations interested in adopting blockchain-based apps will be better informed about the challenges of adoption before implementing them. This helps reduce the perceived risks associated with blockchain-based health apps in health care organizations, as they can develop strategies to overcome the adoption challenges of these apps [114].

### Conclusion

Our study sought to analyze the impact of IT professionals’ perceived risk concerning the intention to adopt BBHAs in health care facilities. Drawing on perceived risk theory, we identified 6 dimensions of risk that may explain IT professionals’ overall risk perception of BBHAs in health care facilities. Data collected from IT professionals in health care facilities through an online survey allowed us to evaluate the hypotheses of our conceptual model. The analysis of these data, using SmartPLS 4 software, yielded important results. The analysis of the correlations between perceived risk and its dimensions revealed that the latter are all positively associated with perceived risk. However, the RDDMP does not contribute significantly to the overall perception of IT professionals’ risks regarding using BBHAs. Then, our results revealed that the significance of the negative association between the general perception of risks of BBHAs and the intention to adopt these apps varies depending on whether the health care organization is in Africa or Canada. This association is nonsignificant in African health care organizations, while it is significant in Canadian health care organizations. Finally, the results of our study revealed that the existence of blockchain solutions in the form of SaaS reduces the overall perception of risks of IT professionals regarding BBHA, given the lack of resources (human and technical) necessary for their use in healthcare structures.

Our study makes the following contributions. First, we highlight the types of risks likely to prevent the adoption of BBHAs in health care organizations despite their interest in adopting these apps.

Second, we show that RDDMP and RLCMD contribute to the overall risk perception of BBHAs. These risks are inherent to the characteristics of blockchain, and the fact that they are not considered in the perceived risk theory makes it unsuitable for risk analysis in blockchain-based apps. The perceived risk theory includes risks that are general to any IT and the integration of these new risks contributes to its extension by making it more suitable for the analysis of risks in blockchain-based apps. Finally, we show that the difference in context between Canada and Africa makes the overall perception of risk of BBHAs differently associated with the intention to adopt these apps between Canadian and African health care organizations. Our findings are beneficial to governments, health care organizations, and blockchain development agencies, which can implement strategies to mitigate some of the risks perceived by IT professionals related to BBHAs.

## Supplementary material

10.2196/77933 Multimedia Appendix 1 Measurement of items.

## Abbreviations

AVE: average variance extracted
BBHA: blockchain-based health care apps
CR: composite reliability
ECBS: existence of cloud-based blockchain solutions
FGBBR: failure to gain blockchain benefits risk
IABHA: adoption intention of blockchain-based health care apps
IBER: immature blockchain ecosystem risk
ORR: organizational readiness risk
PLS-SEM: partial least squares structural equation modeling
PRBHA: perceived risk of blockchain-based health care apps
PRT: perceived risk theory
RDDMP: risk of disclosing data and medical practices
RLCMD: risk of losing control of medical data
SaaS: Software-as-a-Service
SR: security risk
STROBE: Strengthening the Reporting of Observational Studies in Epidemiology
