# Supplementary material for: Resistance to Blockchain Adoption in Health Care Organizations: Evidence From a Cross-Sectional Study
Source: J Med Internet Res. 2026 Apr 8;28:e77933. doi: 10.2196/77933 (PMC13061368; doi:10.2196/77933)
Supplement: Multimedia Appendix 1 [file jmir-v28-e77933-s001.docx]

**Multimedia Appendix 1: Table S1: Items measure** (Items of construct: All items are measured on a continuous 11-point (0–10) semantic differential scale).

| Constructs (8) | Codes | Items |
| --- | --- | --- |
| Risk of disclosing medical practices | I believe that in giving our data and medical practices to other healthcare organizations through blockchain-based applications: | |
|  | **RDMP1** | the risk involved is (very low/very high) |
|  | **RDMP2** | the degree of uncertainty associated is (very low/very high) |
|  | **RDMP3** | the likelihood of expected problems is (very low/very high) |
| Risk of losing control of medical data | **RLCMD1** | The likelihood of having problems in using patients’ medical data is (very low/very high) |
|  | **RLCMD2** | The likelihood of having full control in using patients’ medical data is (very low/very high) |
|  | **RLCMD3** | Healthcare organizations will use patients’ medical data with (no liberty/more liberty) |
|  | **RLCMD4** | The likelihood of consulting and using patients’ medical data without their consent is (very low/very high) |
| Organizational readiness risk | **ORR1** | Technical expertise and knowledge about blockchain in the organization are (unavailable/available) |
|  | **ORR2** | Hesitation to convert to blockchain-based applications is (very low/very high) |
|  | **ORR3** | Tools for implementing blockchain-based healthcare applications in the organization are (unavailable/available) |
|  | **ORR4** | Financial constraints of implementing a blockchain-based application could be (minimal/enormous) |
|  | **ORR5** | New organizational policies for using blockchain technology are (less developed/very developed) |
| Intention to adopt blockchain-based healthcare application | **IABHA1** | The likelihood to continue using blockchain-based healthcare applications in the future is (very low/very high) |
|  | **IABHA2** | The likelihood to always try to use blockchain-based healthcare applications in my daily life is (very low/very high) |
|  | **IABHA3** | The likelihood to plan to continue to use blockchain-based healthcare applications frequently is (very low/very high) |
|  | **IABHA4** | The likelihood of predicting the use of blockchain-based healthcare applications is (very low/very high) |
| Immaturity blockchain ecosystem risk | **IBER1** | Regulation and governance of healthcare blockchain-based applications are (uncertain/clear) |
|  | **IBER2** | Hospitals’ awareness of blockchain technology is (very low/very high) |
|  | **IBER3** | Proportion of hospitals with blockchain-based applications is (very low/very high) |
|  | **IBER4** | Integrability of the blockchain-based applications into the current infrastructures seamlessly is (easy/complex) |
|  | **IBRER5** | Proportion of projects carried out by healthcare organizations aimed at understanding, proving, and testing blockchain-based applications is (very low/very high) |
|  | **IBRER6** | Talent and knowledge acquisition specialized in the development of healthcare blockchain-based applications is (easy/difficult) |
| Failure to gain blockchain benefit risk | **FGBR1** | The likelihood that healthcare blockchain-based applications automate actions and transactions between healthcare organizations is (very low/very high) |
|  | **FGBR2** | The likelihood that healthcare blockchain-based applications lower administrative costs for healthcare organizations is (very low/very high) |
|  | **FGBR3** | The likelihood that healthcare blockchain-based applications lower operational costs for healthcare organizations is (very low/very high) |
|  | **FGBR4** | The likelihood that healthcare blockchain-based applications help in tracking real-time medical data (very low/very high) |
|  | **FGBR5** | The likelihood that healthcare blockchain-based applications increase the efficiency of healthcare organizations is (very low/very high) |
|  | **FGBR6** | The likelihood that healthcare blockchain-based applications enhance the integrity of the healthcare system is (very low/very high) |
|  | **FGBR7** | The likelihood that healthcare blockchain-based applications create an immutable audit trail is (very low/very high) |
|  | **FGBR8** | The likelihood that healthcare blockchain-based applications enhance healthcare systems resilience is (very low/very high) |
|  | **FGBR9** | The likelihood that healthcare blockchain-based applications increase the traceability of healthcare transactions is (very low/very high) |
|  | **FGBR10** | The likelihood that healthcare blockchain-based applications improve regulatory compliance is (very low/very high) |
| Security risk | **SR1** | Transactions in the healthcare blockchain-based applications are (unsafe/safe) |
|  | **SR2** | Healthcare blockchain-based applications have (insufficient security features/adequate security features) |
|  | **SR3** | I perceive the healthcare blockchain-based application as (insecure / secure) |
|  | **SR4** | The decision to use a healthcare blockchain-based application is (risky/safe) |
|  | **SR5** | In general, I believe using a healthcare blockchain-based application is (risky/safe) |
|  | **SR6** | Patients’ medical data is (insecurely managed in the healthcare blockchain-based application/securely managed in the healthcare blockchain-based application) |
|  | **SR7** | Healthcare blockchain-based application is (unsafe for patient’s medical data/safe for patient’s medical data) |
| Existence of blockchain-based healthcare applications as cloud applications (moderating effect) | **EBHACA** | The existence of blockchain-based healthcare systems usable from the cloud (cloud computing) reduces healthcare professionals’ perception of risk regarding the lack of resources (human, technical and financial) required for the adoption of blockchain-based healthcare systems in hospitals (strongly disagree/strongly agree) |
